# Household cost of accessing contraceptive services among women in Urban communities in Ghana

**DOI:** 10.1371/journal.pone.0325882

**Published:** 2025-06-10

**Authors:** Caesar Kaba Kogoziga, Desmond Dzidzornu Otoo, Raphael Kwasi Gborgbortsi, Richmond Owusu, Serwaa Akoto Bawua

**Affiliations:** 1 Department of Health Policy Planning and Management, School of Public Health, University of Ghana, Legon Accra, Ghana; 2 Department of Biological, Environmental, and Occupational Health Sciences, School of Public Health, University of Ghana, Legon Accra, Ghana; National Institute of Public Health: Instituto Nacional de Salud Publica, MEXICO

## Abstract

**Background:**

In many developing nations, including Ghana, access to contraceptive services, remains a critical concern where urban areas face unique challenges in healthcare delivery. Despite various interventions, the financial burden of assessing these contraceptive services continues to hinder adoption by women especially those with economic challenges. This study explored the costs incurred by women seeking contraceptive services in urban communities by estimating the direct, indirect, and intangible costs in Ghana.

**Methods:**

A facility-based cross-sectional study was conducted using the patient perspective; to gather data on direct medical and non-medical costs, indirect costs and intangible costs that were associated with women seeking contraceptive services. A structured questionnaire was used to collect data from three Planned Parenthood Association of Ghana (PPAG) facilities in the Accra metropolitan, Suame municipal and Sagnarigu districts in the Greater Accra, Ashanti, and Northern Regions respectively. A total of 125 women accessing contraceptive services were randomly selected and included in the study. Data was analyzed descriptively and reported in frequency tables, pie, and bar charts. All costs were reported in Ghana Cedi and US dollar.

**Results:**

The average direct cost of contraceptive services was GHS 18.37 ± 22.11 (US$ 1.53 ± 1.84) per visit. This comprised an average direct medical cost of GHS 8.50 ± 7.18 (US$ 0.71 ± 0.60) and non-medical cost of GHS 9.84 ± 20.23 (US$ 0.82 ± 1.69). Clients, on average, lost 52.1 minutes due to traveling and waiting, resulting in an average productivity loss of GHS 1.62 per visit. The average economic cost of contraceptive service was GHS 19.99 (US$ 1.67) per patient. About 92% of the economic cost was made up of direct cost. 71.2% of respondents consulted their partners before accessing contraceptive services, and 94% believed that their decision to use contraceptives did not negatively affect their relationships, however, many reported pains during the procedure.

**Conclusion:**

The study highlights the considerable direct and indirect costs associated with accessing modern contraceptive services, indicating a potential barrier to access when compared to daily minimum wage and prevailing economic conditions. Addressing these economic challenges is crucial for ensuring access to contraceptive services. Innovative strategies such as service delivery outreaches and deployment of digital health interventions to expand self-care is recommended to help reduce travel time to and from the service delivery point for contraceptive services.

## Introduction

Family planning (FP) and access to contraceptive services remain a concern in several developing countries [1]. Access to contraceptive services is an essential component in empowering women, enabling them to make informed decisions about their reproductive health. The average number of children born to women during their reproductive years, is one of the most important factors in population growth. High fertility rates are closely linked to poverty [2], poor maternal and child health outcomes [3], and slow economic development [1]. There is a global consensus that countries across the world must continue to work aggressively towards achieving a relatively low fertility rate to propel economic growth and eliminate poverty, and a lot of investment is needed to achieve this globally [4]. Access to contraception provides a good opportunity to empower women and adolescent girls, increase investments in children, and ultimately contribute to poverty reduction [5,6]. Improved access to contraceptives is critical to achieving improved maternal health, reducing child mortality, and combating HIV/AIDS [7]. Limited access to contraceptive services is a major contributor to unintended pregnancies [8], unsafe abortions, and complications during childbirth [9], which collectively impose a significant burden on healthcare systems and households.

In a developing country like Ghana, the need to manage fertility rate is even more urgent. Usage of contraceptives has a significant effect on the health system as it helps to prevent unintended pregnancies, reduce the number of abortions, and lower the incidence of death and disability related to complications of pregnancy and childbirth [4]. Studies suggest that access to modern contraceptives in developing countries could reduce the problem of unmet need for contraceptive uptake and reproductive health services, avert over 54 million unwanted pregnancies and 26 million abortions worldwide each year [10]. Attempts to increase the number of FP users have been plagued with several barriers within various dimensions, such as accessibility, availability, awareness, and affordability of services [10]. Cost remains a significant barrier to contraceptive use among several group of people in Ghana especially among young women [11]. Despite the significant efforts in Ghana targeted at integrating FP services into other health services [12], ensuring continuous supplies of a broad range of contraceptive methods, and building the capacity of service providers, unmet need for contraceptives is still high (30%) in Ghana [13].

Improving FP services and contraceptive uptake coverage in low-middle-income countries are topical public health concerns. Urban areas, marked by their concentrated populations and diverse demographics, often present unique challenges regarding healthcare access. Within these urban communities, women, in particular, encounter multifaceted barriers to accessing essential contraceptive services [14]. Cost of accessing contraceptive devices is a major barrier in most developing economies including Ghana where healthcare financing is heavily reliant on out-of-pocket payment for certain health services [15]. The cost associated with contraceptive devices may present a significant challenge to individuals extending to their households.

Ghana’s revised National Health Insurance Act, 2012 (Act 852) stipulates for the inclusion of contraceptive services to the National Health Insurance Scheme (NHIS) benefit package. However, implementation of this policy has not seen much progress, and contraceptive services continue to be paid out-of-pocket in most public and private facilities across the country [16]. Due to the unequal distribution of health services across the country, consumers of contraceptive services experience different levels of cost to access contraceptive services, including travel time, waiting time, loss of or suspension of productive time/activities [15]. The level of indirect cost, in the context of the consumer, has a direct influence on their ability to access services. Amissah et al., [15] found that contraceptive services are unequally distributed across the country and clients travel a long distance to access contraceptive services especially in urban areas. However, little is known about the cost burden of accessing FP services among women in urban parts of Ghana. Although Amissah et al.’s study [15] provided a comprehensive analysis of FP service costs in Ghana, this study focused specifically on urban areas, which often face distinct challenges in accessing health services compared to rural. These may include denser population, increased transportation costs [17] and diversity of health seeking behavior [18]. This study also introduces aspects of intangible costs which have not been extensively covered by other studies. Understanding intangible costs adds value by providing insight into the emotional and social barriers that may deter individuals from accessing contraceptive services. These barriers can impact decision-making and sustained contraceptive use, even when financial and physical access barriers have been tackled [19]. Hence, this paper estimates the household cost including intangible costs of accessing modern contraceptive services among women in urban communities in Ghana.

## Materials and methods

### Study design and population

The study employed a facility-based cross-sectional design using quantitative approaches to estimate household cost of accessing contraceptive services. A patient perspective was used in the cost analysis. The study included women aged 18 years and above accessing contraceptive services in the selected Planned Parenthood Association of Ghana (PPAG) facilities within the study period Women who were very ill, mentally incapacitated and those who visited for other services other than contraception were excluded.

### Study area

The study was conducted in three [1] urban Metropolitan Municipal and District Assemblies (Accra Metropolitan, Suame Municipal and Sagnarigu district) across the three ecological zones of Ghana – Coastal belt, Middle belt and Savannah. The respective regions were Greater Accra, Ashanti, and Northern Regions of Ghana. These districts were selected because of the existence of Planned Parenthood Association of Ghana (PPAG) facilities providing contraceptive services. A total of three [1] PPAG facilities were selected with one from each of the three districts. PPAG is the leading, rights-based NGO in the field of Sexual and Reproductive Health and Rights (SRHR) in Ghana.

### Sample size estimation

The sample size was estimated using the single sample for an infinite population formula. A similar study conducted by [15] average household expenditure was estimated to be GHS 25.67 with a standard deviation of GHS 5.7.


$$Sample\;Size\;(N)=\frac{\left(z^{2}\times \sigma^{2}\right)}{e^{2}}$$

Where: n = sample size; Z is the critical value (1.96) of 95% confidence level.

σ is the Standard deviation (GHS 5.7)

 e is the margin of error estimated at GHS 1.05 (4% of estimated household expenditure)


$$N={\left(\frac{1.96\;\times 5.7}{1.05}\right)}^{2}=114$$

Accounting for 10% non-response rate = 114 x 0.1=11. 


Hence the minimum sample was 114+11=125

### Sampling procedure

A multi-Stage sampling technique was used to recruit 125 women seeking contraceptive services in the selected facilities. In the first stage, the three [1] urban districts across 3 different regions in Ghana were conveniently selected to represent the southern, middle, and northern belts. In the second stage, the Accra metropolitan, Suame municipality and Sagnarigu districts in the Greater Accra, Ashanti, and Northern Regions respectively were purposively selected because of the existence of PPAG facilities providing contraceptive services.

In the third stage, a simple random sampling technique was used to recruit clients at the facility level. To ensure that all members of the population had an unbiased and equal chance of selection, the balloting method was employed. Pieces of paper were prepared, each bearing either a ‘yes’ or ‘no’. Women who walk into the facility to access services were requested to pick a paper from a container. Clients who picked ‘yes’ and consented to participate were included in the study, while those who picked ‘no’ were excluded. This procedure was repeated until we reached our minimum sample size. 41 women from Accra, 35 from Suame and 49 women from Sagnarigu were included in the study.

### Data collection tools and procedures

A structured questionnaire developed in line with existing literature [15] was used for data collection through a face-to-face interview approach. The questionnaire was designed electronically on Kobo Collect toolbox [20] and administered on a smartphone by trained Resident Enumerators (RE) with assistance from health care professionals on duty. The questionnaire included key questions on direct, indirect, intangible costs and sources of finance. The questionnaire was pre-tested at PPAG Family Health Clinic in Accra for one day, and the feedback from the pre-testing was used to revise the tool to make it more effective. During the pre-testing, eighteen [18] interviews were done with clients (six interviews per zone). The REs were trained in the use of the Kobo Collect application for data collection and translation of the questions into the different languages.

During data collection, eligible participants were taken through the participants information individually and a written informed consent was taken before data collection. All questions were asked in English, and where necessary, in the respondent’s preferred local dialects (Twi, Ga, and Dagbani). The entire process of recruitment and data collection took place between April to June 2023 in the 3 selected facilities.

### Data processing and analysis

A cost of illness approach focusing on direct cost and lost time value were adopted to estimate the cost of accessing contraceptive services in urban communities. This method has widely been adopted in estimating the cost of under-reported public health issues in several studies [15]. Data was organized using Microsoft Excel and STATA version 16 and made ready for analysis. Means, standard deviation, frequency, and proportions were used to summarize data. All estimates were expressed in Ghana cedis (GH₵) and US Dollar (US$). An average exchange rate of US$ 1 = GHS12 as at November 2023 was used in all estimations. A daily minimum wage of GHS14.88 for 2023 in Ghana was used in discussion [21]. This was used to provide a standardized comparison that reflects the minimum income required for daily sustenance.

### Cost estimation

#### Estimating direct cost of accessing contraceptive services.

The term “direct costs” refers to expenses incurred as a direct result of accessing contraceptive services, such as consultation fees, tests, and patient transportation. Direct cost was estimated as medical cost and non-medical cost. The direct cost was estimated using approaches adopted by Amissah et al., [15] on the cost incurred by clients in seeking FP services at the service delivery points (SDP). It was estimated by summing up all costs incurred by clients seeking contraceptive services at the SDP.

**Direct cost** = folder/card fee + consultation fee + commodity purchased + transportation cost.

#### Estimating the indirect cost of accessing contraceptive services.

Indirect costs were estimated through the reported lost time by clients. The time value lost is estimated on the assumption that every time lost owing to contraceptive service contributes to productivity losses.

**Indirect Cost (Time value loss) =** summation of traveling time + distance traveled + waiting time at SDP for FP services.

#### Estimating economic cost.

The economic cost of contraceptive services was the total resources required to access contraceptive services. This included direct costs as well as indirect costs.

Economic Cost = Total direct cost + Total indirect costs

#### Estimating intangible cost.

Intangible costs refer to non-monetary expenses associated with care provision, including psychological and social factors such as pain, anxiety, fear, stigma, or the impact on social relationships. These costs are subjective and vary among individuals, making them challenging to quantify [22]. Intangible cost was estimated by measuring the level of pain and anxiety associated with accessing contraception using the Numerical Rating Scale (NRS), as well as the impact of the client’s decision to access contraception on their relationships. The measurement of pain, anxiety, and the impact on a relationship is inherently subjective. Different individuals may interpret and rate their experiences differently, making it challenging to obtain objective and standardized data.Pain was graded as 0 = No pain; 1–3 = mild; 4–6 = moderate; 7–10 = severe. Table 1 summarizes the different types of costs assessed in the study and their cost components.

**Table 1:** Summary of costs and cost components.

Type of cost	Category of Cost	Components
Direct cost	Medical	• Cost of consultation • Cost of commodity • Cost of other contraceptive services (e.g., removal of implant)
	Non-Medical Cost	• Cost of transportation • Cost of feeding
Indirect cost	Productivity losses	• Productivity loss to patient • Travelling time • Client waiting time
Intangible cost	Psychological	• Associated Pain • Anxiety/fear
	Social Relationship	• Partner’s acceptance • Effect on relationship

### Ethical consideration

Ethical approval for the study was sought from the Ghana Health Service (GHS) Ethics Review Committee (ERC). Privacy and confidentiality were upheld during the research. Participants were given adequate information about the study and written informed consents were obtained before data collection. All Covid-19 protocols were upheld during the study.

## Results

### Sociodemographic characteristics of study participants

A total of 125 participants were included in the study. Table 2 below shows the sociodemographic characteristics of study participants. Out of the total number of respondents, the majority (81.6%) were above the age of 20 years while the rest were between 18 to 20 years. Half (50.4%) were married whiles 41.6% were single whiles the rest were cohabiting (7.2%) or separated (0.8%). In terms of education, 37.6% had at least senior high level of education, 25.6% had junior high level of education while 11.2% had some form of tertiary education. 6.4% had no form of formal education. In relation to employment status, the majority (44%) of the participants were self-employed while 34.4% were unemployed. The rest were into other forms of full-time (16%) and part-time (5.6%) employment. More than half of the respondents were Muslims (65.6%) whiles 33.6% were Christians. As shown in Table 2 below, in the last 12 months, 52% of the respondents had visited the service delivery point once for contraceptive services, 16.8% visited two times, 3.2% visited three times, and 28% of the respondents visited four times. About 60 (48%) of the respondents reported that the cost of assessing contraceptives was financed by their spouse. 53 (42.4%) financed the cost by themselves while the rest had other sources of finance. Approximately 89 (71.2%) of all respondents indicated that they consulted their partners before taking a decision to access contraceptive services while the rest did not consult their partners. (Table 2).

**Table 2:** Socio-demographic characteristics of participants.

Variables	Frequency (n = 125)	Percentage (%)
**Age groups (years)**		
18 - 20 years	23	18.4
Above 20 years	102	81.6
**Marital status**		
Cohabiting	9	7.2
Married	63	50.4
Separated	1	0.8
Single	52	41.6
**Educational Level**		
None	8	6.4
Primary	6	4.8
JHS/JSS	32	25.6
SHS/Tech	47	37.6
Tertiary	14	11.2
Other	18	14.4
**Employment status**		
Full time	20	16
Part-time	7	5.6
Self-employed	55	44
Unemployed	43	34.4
**Religion**		
Christian	42	33.6
Muslim	82	65.6
**Number of visits in the last 12 months**		
Others	1	0.8
Once	65	52
Twice	21	16.8
Three times	4	3.2
Four times	35	28
**Sources of finance support**		
Others	12	9.6
Self	53	42.4
Spouse	60	48
**Consultation with partner before using contraception**		
No	36	28.8
Yes	89	71.2

### Choice of contraceptive method

**Fig 1:**
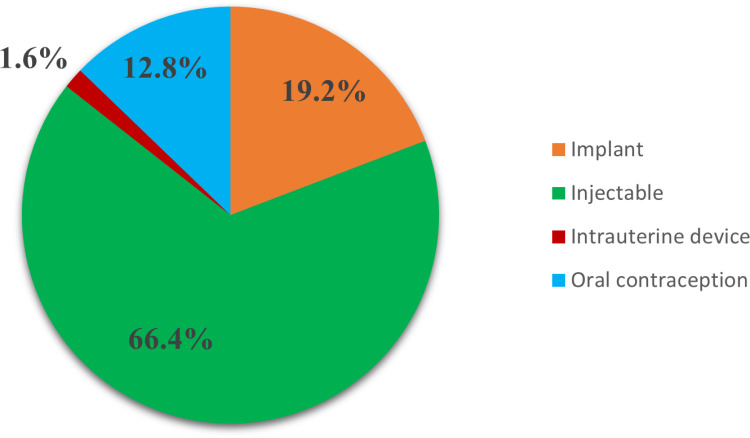
Choice of contraceptive methods.

In terms of contraceptive preference, more than half (66.4%) of respondents opted for injectables, 19.2% preferred intrauterine devices (IUDs), 12.8% chose oral contraceptives, and 2.4% preferred an implant. This distribution is captured in Fig 1 below (Fig 1).

### Direct cost of contraceptive services

Table 3 summarizes the estimation of direct cost of contraceptive services. The average direct cost of contraceptive service was GHS18.37 ± 22.11 (US$ 1.53 ± 1.84) per patient with median of GHS9.5 (IQR:7–14). This comprised an average direct medical cost of GHS8.50 ± 7.18 (US$ 0.71 ± 0.60) per patient with median of GHS7 (IQR: 4–12) and non-medical cost of GHS9.84 ± 20.23 (US$ 0.82 ± 1.69) per patient with median cost of GHS3(0–5). Direct non-medical costs accounted for 53.6% of the cost of contraceptive services while 46.4% was attributed to direct medical costs. Cost of commodities (contraceptive) accounted for 58.6% of the total direct medical costs and 27.2% of the total direct costs. 77.7% of the total direct non-medical costs and 41.7% of the total direct costs were due to the cost of transportation (Table 3).

**Table 3:** Direct cost of assessing contraceptive services.

Cost item	Cost GH₵	Cost USD	Average cost GHS	Median (IQR)	Average cost USD	Cost profile (%)
**Direct medical cost**						
Cost of consultation	441	36.75	3.53 ± 4.49	2 (1,3,4)	0.29 ± 0.37	19.2
Cost of commodity	625	52.08	5.00 ± 4.79	4 (1,-6)	0.42 ± 0.40	27.2
**sub-total**	**1,066**	**88.83**	**8.53 ± 7.18**	**7 (412)**	**0.71 **± 0.60	**46.4**
**Direct non-medical cost**						
Cost of food/Snack/water	124	10.33	0.99 ± 4.76	0 (0-0.0)	0.08 ± 0.40	5.4
Cost of transportation	956	79.67	7.65 ± 14.28	3 (0-5)	0.64 ± 1.19	41.7
Other payments	150	12.50	1.20 ± 7.68	0(0−0)	0.10 ± 0.64	6.5
**Sub-total**	**1,230**	**102.50**	**9.84 **± 20.23	**3(0-5)**	**0.82 **± 1.69	**53.6**
**Total direct cost**	**2,296**	**191.33**	**18.37 **± 22.11	**9.5(714)**	**1.53 **± 1.84	**100**

### Average exchange rate of US$ 1 = GHS 12 as at November 2023 was used in all estimations

The study further estimated the costs of various contraceptive methods. As shown in Table 4 below, Intrauterine devices are associated with the highest average cost of GHS 48.2 (US$ 4.02) per patient, while oral contraceptives had the least average cost of GHS 10.9 (UD$ 0.91) per patient. The total cost of the contraceptives among all participants was GHS 2,763.8 (US$ 230.32). Considering the contraceptive preferences of the 125 participants, the cost of injectables was GHS 1842.6 (US$ 153.55), which accounted for 66.67% of the total costs. Intrauterine devices (least preferred) had a cost of GHS 96.40 (UD$ 8.03) and accounted for 3.49% of the total cost of contraceptives. (Table 4).

**Table 4:** Cost distribution per choice of contraceptive method.

Contraceptive method	Minimum GHS(USD$)	Maximum GHS(USD$)	Average GH₵	Average US$	Total cost GH₵	Total Cost (US$)	Cost profile (%)
Implant	7.90 (0.66)	109.9 (9.16)	27.1	2.26	650.4	54.20	23.53%
Injectable	4.60 (0.38)	107.3 (8.94)	22.2	1.85	1842.6	153.55	66.67%
Intrauterine device	32.10 (2.68)	64.4 (5.37)	48.2	4.02	96.4	8.03	3.49%
Oral contraception	7.60 (0.63)	15.7 (1.31)	10.9	0.91	174.4	14.53	6.31%
**Total**	**52.20 (4.35)**	**297.30 (24.78)**	**108.40**	**9.03**	**2763.8**	**230.32**	**100.00%**

### Indirect cost of accessing contraceptives

Regarding indirect cost, clients lost an average of 52.13 minutes owing to traveling and waiting time. Using the minimum wage of GHS 14.88 (US$ 1.24) per day for a period of 8hours of work a day, the minimum wage for an hour of work was GHS 1.86 (US$ 0.16). From Table 5, participants lost a total of GHS 113.93 (UD$ 9.49) to travel time which accounted for 56.32% of productivity loss. A total of GHS 88.35 (US$ 7.36) was lost due to waiting time in the facilities, which accounted for 43.68% of total productivity loss. Averagely, participants lost GHS 1.62 (US$ 0.13) due to travel and waiting time. (Table 5).

**Table 5:** Indirect cost associated with accessing contraceptive services.

Cost item	Average Time Loss Mins(Hrs.)	Total Cost of time loss GHS	Total Cost of time loss US$	Average cost GHS	Average cost US$	Cost profile (%)
**Productivity loss due to time travel**	29.3 (0.49)	113.93	9.49	0.91	0.08	56.32
**Productivity loss Due to waiting time**	22.9 (0.38)	88.35	7.36	0.71	0.06	43.68
**Total**	52.1 (0.87)	202.28	16.86	1.62	0.13	100

### Economic cost of contraceptive services

Table 6 shows the total economic cost of accessing contraceptive services. The average economic cost of contraceptive service was GHS 19.99 (US$ 1.67) per patient. The majority (91.90%) of the economic cost was made up of direct cost, while the remaining (8.10%) was indirect cost. The cost of transportation accounted for 38.27% of the total economic cost followed by the cost of commodity (contraceptive) accounting for 25% of the total economic cost. Productivity loss due to waiting time was the least contributor (3.54%) to the total economic cost. (Table 6).

**Table 6:** Economic cost of assessing contraceptives.

Cost item	Cost GHS	Cost US$	Average Cost GHS	Average Cost US$	Cost profile (%)
**Direct cost**					
Cost of consultation	441	36.75	3.53	0.29	17.65
Cost of commodity	625	52.08	5.00	0.42	25.02
cost of food/Snack/water	124	10.33	0.99	0.08	4.96
Cost of transportation	956	79.67	7.65	0.64	38.27
Other Payments	150	12.5	1.20	0.10	6.00
**Sub Total**	**2,296**	**191.33**	**18.37**	**1.53**	**91.90**
**Indirect cost**					
Productivity loss due to time travel	113.93	9.49	0.91	0.08	4.56
Productivity loss Due to waiting time	88.35	7.36	0.71	0.06	3.54
**Sub Total**	**202.28**	**16.85**	**1.62**	**0.14**	**8.10**
**Total economic cost**	**2,498.28**	**208.18**	**19.99**	**1.67**	**100**

### Intangible cost

**Fig 2:**
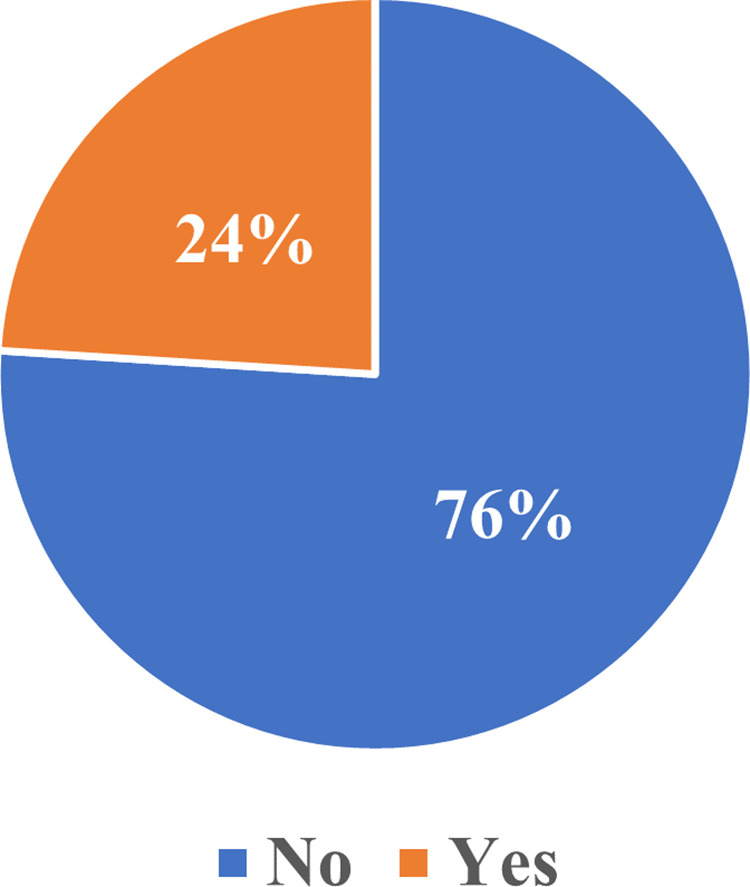
Fear associated with contraception use.

**Fig 3:**
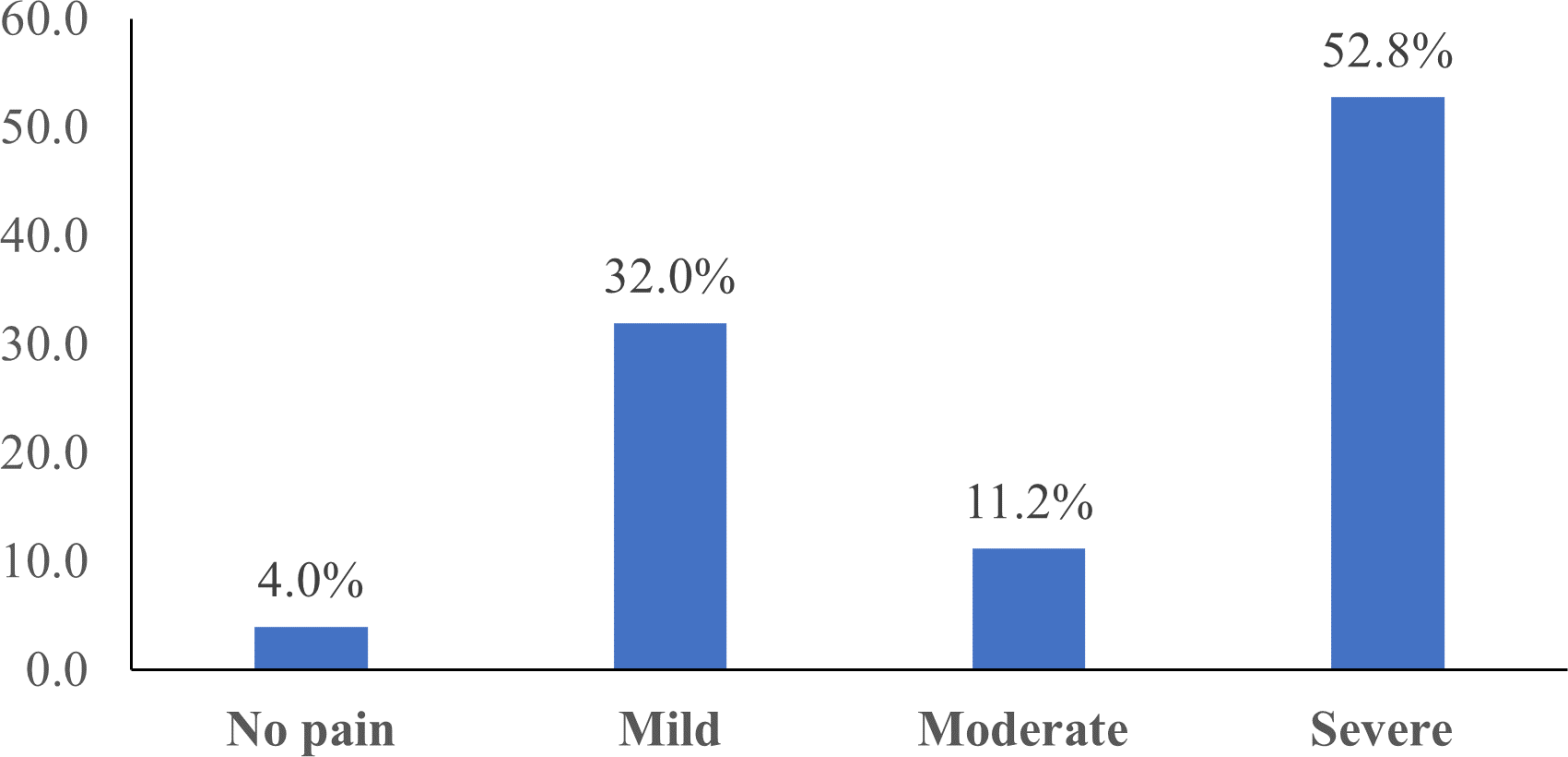
Pain associated with the procedure.

Regarding fear and anxiety associated with accessing contraceptives, 76% reported that they had no fear associated with using the contraceptive (Fig 2). In terms of Pain associated with the procedure, 52.8% reported severe pain, 11.2% reported moderated pain, 32% reported mild pain, and 4% reported no pain associated with the use of contraceptives (Fig 3).

**Fig 4:**
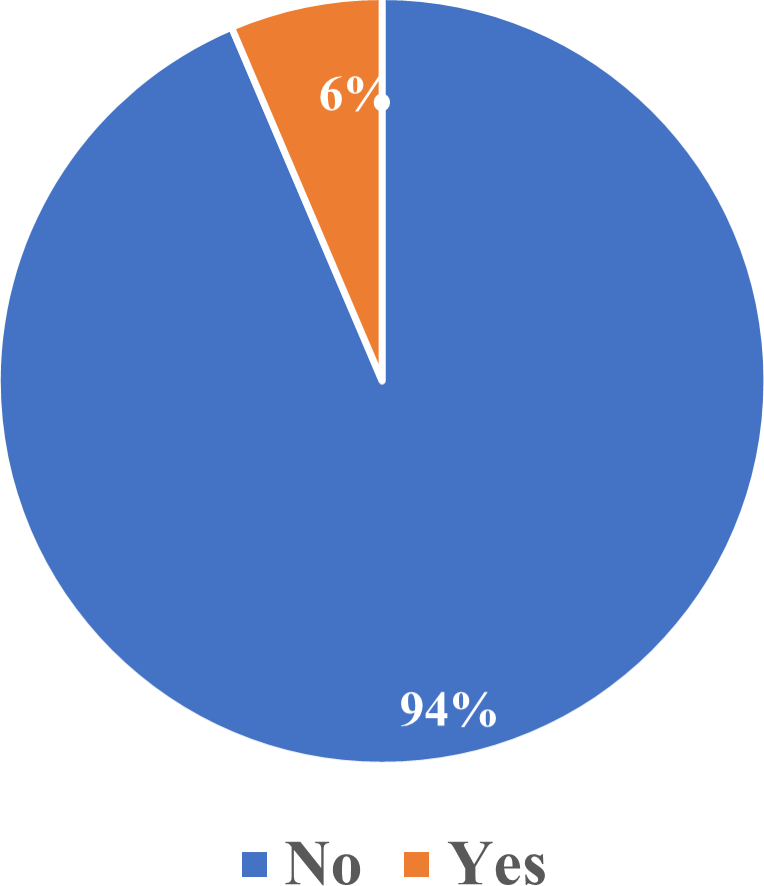
Decision to use contraception affecting relationships.

In terms of their social relationships, 94% also indicated that their decision to access contraception will not have a negative effect on their relationship (Fig 4).

## Discussion

In the effort to increase access to contraceptive services, clients, service providers, and government policy implementers must consider how the direct cost of contraceptive services (including out-of-pocket expenses and transportation costs) and their affordability directly influence women’s ability to access services or not. The findings from this study showed that clients incurred an average direct cost of GHS 18.37 ($1.53) in accessing contraceptive service. This finding is higher than the costs reported in a study in Ghana [15] where clients spent an average direct cost of GHS 7.90 ± 5.7 ($1.76 ± 1.27) when accessing FP services. Although the study was published recently, the year of data collection (2017/2018) could account for the lower costs reported as compared to this study. The differences in cost may be attributed to inflation and changes in the cost of living over time, since this current study collected data in 2023, while the earlier study used data from 2017/2018. According to Ghana’s Consumer Price Index (CPI), the average inflation rate by end of 2018 was 9.4% [23] compared to 23.2% [24] by end of 2023. This inflation likely contributed to the observed increase in direct costs over the period. Cost finding in our study is relatively high when compared to the daily minimum wage of the Ghanaian working population (GHS 14.88) in 2023. A similar study conducted under the health policy project in Ghana [25] found that the average cost per client per year for assessing FP services was between GHS 135.29 (US$ 75.16) and GHS 82.60 (US$45.89) for female condoms and combined injectable contraceptive (CIC) to GHS 11.75 (US$6.53) and GHS 11.25 (US$ 6.25) for natural family planning (NFP) and lactational amenorrhea method (LAM), respectively [25]. The health policy project [25] involved a larger number of facilities and measured the cost over a year whiles this study considered the costs associated with accessing FP services per session. The methodological and contextual differences highlighted above could be a reason for the cost differences between the two studies. However, for some contraceptives like the injectables, the cost of GHS 22.2 (US$ 1.85) per session when aggregated annually, will be close to the higher annual costs reported by the health policy project [25]. Similar findings were reported in Kenya where out-of-pocket payments of US $0.91 were made for injectables and US$ 4.31 for implant users [26]. The cost of injectables in Kenya was lower than reported in Ghana (US$ 1.85) in this current study although the cost of implants was higher in Kenya than in Ghana (US$ 2.26).

In Latin America, it was reported that women spent about US$ 1 per month out-of-pocket on contraceptives [27]. A 2016 study in the United States reported a mean total out-of-pocket expenses for permanent contraceptives as US$ 82 and non-permanent contraceptives as US$ 30 in 2013. For oral contraceptives, an amount of US$ 26 was made out of pocket whiles US$ 20 was the mean total out of pocket payment for intrauterine devices [28]. Compared to the study in a higher income country such as the United States, although more was spent on contraceptives than in this current study, it is important to consider local income levels and economic vulnerability. The burden in Ghana may be higher since average cost exceeds the daily minimum wage.

Findings of this study show that consumers on daily minimum wage (GHS 14.88) spend about 19% more of their wage on direct costs which is estimated at GHS 18.37 per session to receive contraceptive services. This highlights the financial burden placed on individuals with lower incomes who may need more than their daily earnings to access contraceptive services. Evidence from other studies suggests that healthcare funding strategies that heavily emphasize out-of-pocket expenses can cause household poverty [13]. There exists increasing evidence that households are forced into catastrophic expenditure or are forced into greater poverty because of high healthcare costs [29]. Contraceptive services are severely hampered by the comparatively high direct cost of contraceptives in relation to the daily minimum wage, which creates a heavy financial burden on the household budgets of the average Ghanaian. The Sustainable Development Goal (SDG) three emphasizes that households will be experiencing catastrophic health expenditure if household expenditure on health exceeds 10% [30].The higher than daily minimum wage cost of contraceptive access has the tendency to deter clients with low incomes from obtaining routine care and eventually leads them to opt for other money-saving choices. Users of modern contraceptives might be pressured in this situation to turn to ancient methods, which have a high failure rate [31], and this hinders global efforts to increase women’s control over their fertility. Although in this study, several women spent more than the daily minimum wage on contraceptives, a study in South Africa on the cost of assessing sexual and reproductive health services reported that very few women experienced catastrophic health expenditure and this was for infertility [32].

In Ghana a parliamentary Act 650 created the National Health Insurance Scheme (NHIS) in 2003 to provide financial risk protection from the cost of healthcare services. Contraception was not included in the original design, despite the generous benefit package [29]. Evidence suggests that the cost of contraceptives is a major barrier to access for many women in low- and middle-income countries like Ghana [31]. Thus, the high direct cost estimated by Darroch et al. [33] suggest that there is a tendency to alleviate financial barriers if various forms of contraceptive methods are offered to NHIS covered customers in Ghana following the full operationalization of the extended package of NHIS which adds FP services to the NHIS benefit package [34]. This will ensure the elimination of user fees and differential payments for FP methods.

In addition, the average direct cost comprised of GHS 8.53(46.4%) of direct medical cost and GHS 9.84 (53.6%) of direct non-medical cost. The direct medical cost is influenced by the choice of contraceptive, for instance, intrauterine devices are associated with the highest average cost (GHS 48.2), while oral contraceptives have the least average cost (GHS 10.9). Injectables and implant made up 22.2% and 27.1% respectively. This shows that the long-acting contraceptives (Intra Uterine Device and implant) were associated with higher direct cost as compared to the short acting methods (Injectables and oral pills). Similar findings were noted in Kenya [26] and the United States [28,35] where costs of long-term contraceptives were higher as compared to short term ones. According to research, the cost-effectiveness of long-term contraceptive techniques is higher. As a result, purchasing long-term contraceptives would result in cost savings despite a higher initial service delivery cost [36].

As part of the direct non-medical costs, the cost of transportation alone constituted 41.7% of the total direct cost of accessing contraceptive services. This suggests that, even if the medical cost of contraceptive services is absorbed through the NHIS, the direct non-medical cost, especially, transport cost may still serve as a barrier to accessing contraceptive services among Ghanaian women. This can be associated with the distance to and from the service delivery point. That notwithstanding, if contraceptive products are made free it will still benefit women who need contraceptives and reduce their cost burden. A study among women in Tanzania reported that women who paid 2,000 Tanzanian shillings and higher for transportation to access FP services had lower usage of modern methods compared to those who paid nothing [37]. Thus, the finding of the Tanzania study was similar to our finding. It is important to note that the current study did not include non-users as such although high cost of transportation may impede contraceptive use among women it may further studies may be needed to determine the significance of transportation cost as a barrier.

Indirect costs refer to other losses incurred such as lost wages, lost productivity, and costs that would otherwise not be incurred. Consumers of contraceptive services incur varying degrees of indirect costs to receive contraceptive services due to the uneven distribution of health services across the nation, including travel time, waiting time, loss of or interruption of productive time/activities, and travel time. The amount of indirect costs in the consumer’s situation directly affects their ability to receive services.

According to the study’s findings, customers lost an average of 52.13 minutes each visit due to travel time (29.3 minutes) and waiting time (22.9 minutes). The client’s lost productivity time may result from the lengthy journey time to health care facilities. Clients must pay a minimum of GHS 1.62, or approximately 11% of the Ghanaian minimum wage, for the rather lengthy travel and waiting time. This is less than the projected time lost in a study of a similar nature [15], which found that users of financial planning (FP) services lost a total of 73 minutes on each visit to a service delivery point (SDP) due to travel and waiting time. This implies that eliminating the direct cost of contraception alone may not be effective in increasing uptake if strategic interventions are not put in place to reduce the indirect cost of accessing contraceptives.

Economic cost is both the explicit cost and the opportunity cost. This cost includes the gains and losses in terms of money, time, and resources. It considers both the monetary value and the choices not selected because of the choice selected. There is increased focus on the economic costs of health care, as demand for health care outstrips available resources. To the client, economic cost is key determinant of the accessibility of health services, including contraceptive services [38]. As seen in this study, the average economic cost of contraceptive service is GHS 19.9. The majority (92%) of the economic cost is made up of direct costs, while the remaining (8%) is indirect cost. According to this study, the economic cost of contraceptive services is relatively high when compared to the daily minimum wage of the Ghanaian working population (GHS 14.88) in 2023 [21]. This shows that FP clients spend more to receive contraceptive services than the nation’s daily minimum wage (daily income). This means that FP clients must sacrifice more than one day of their wages to access contraceptives. The national daily minimum wage used provides a standardized comparison that reflects the minimum income required for daily sustenance. While this study focused on urban populations, who are likely to have higher average incomes than the national average, the minimum wage highlights the economic strain that accessing contraceptive services may impose on lower-income or economically vulnerable individuals in the urban areas. According to the results of this study, 34.4% of all the respondents were unemployed. Also, 42.4% of these respondents finance the cost of contraceptives themselves. Relating this to the economic cost of contraceptives, these unemployed respondents may find expenditure on short-term contraceptives catastrophic, as they have little or no source of income. However, these costs may be acceptable for long-acting contraceptive methods since the cost incurred may provide coverage for 3–5 years. To holistically solve this problem, the extension of the National Health Insurance to cover cost of modern contraceptive methods in Ghana [39] is timely and should be operationalized fully as soon as possible to relieve the burden on users.

Intangible costs are those associated with function loss, increased pain, and reduced life quality. Intangible costs are defined as pain, anxiety, stigmatization associated with accessing contraceptive services and its impact on social relationships, which are usually measured by using the reduction in quality of life. This is because the procedure, depending on the method, may sometimes be invasive or involve an injection.

### Limitations

This study was limited to just three [1] urban districts in Ghana, in three [1] Planned Parenthood Association of Ghana (PPAG) facilities, who were selected based on a convenience sampling technique. Findings of this study applies to urban clinic users and may differ for rural populations or non-clinic settings. Costs in this study might be different in government clinics or for women who never reach services. The costing tool was self-created but guided by available literature. Nevertheless, the facilities selected had the potential to represent the northern, middle, and southern belts of the country. Several scientific checks were used, including pre-testing of the tools, a random selection of respondents, and the creation of tools based on past literature reviews of the standard costing questionnaire, and discussions of the findings with pertinent literature. Thus, the tool captured all significant costs associated with accessing contraceptive services.

## Conclusion

The costs incurred by households in accessing contraceptive services in this study exceed the national daily minimum wage for many users. This poses potential barriers to access, particularly for marginalized populations.

Costs of transportation and productivity losses were major contributors to the economic burden. Thus, it is important to improve geographical access by bringing services closer to the people in communities. While more than half of the women experienced severe pain associated with the use of contraceptives, it did not deter them from continuous use or harm their relationships. Intangible costs such as stigma and anxiety were not major barriers as anticipated with high partner involvement highlighting a shared decision making for FP services.

Addressing these economic barriers to contraceptive access is essential for promoting equity. Policymakers and stakeholders should consider interventions aimed at reducing financial burdens such as fully implementing the extended package for FP on the NHIS, streamlining service delivery, improving access to services locally and fostering an environment conducive to accessible and affordable contraceptive services for all. Innovative strategies including service delivery outreaches and the deployment of digital health interventions to expand self-care products are greatly encouraged to help reduce travel time to and from the service delivery point for contraceptive services.

## List of abbreviations:

FP: Family Planning
PPAG: Planned Parenthood Association of Ghana
NHIS: National Health Insurance Scheme
SDP: Service Delivery Point
SRHR: Sexual and Reproductive Health and Rights
IUD: Intrauterine Device
